# Hymeglusin Enhances the Pro-Apoptotic Effects of Venetoclax in Acute Myeloid Leukemia

**DOI:** 10.3389/fonc.2022.864430

**Published:** 2022-06-29

**Authors:** Cheng Zhou, Zhiqin Wang, Shuanghui Yang, Huan Li, Liang Zhao

**Affiliations:** ^1^ Department of Hematology, Xiangya Hospital, Central South University, Changsha, China; ^2^ Department of Geriatric Neurology, Xiangya Hospital, Central South University, Changsha, China; ^3^ Department of Oncology, Xiangya Hospital, Central South University, Changsha, China

**Keywords:** AML, venetoclax, hymeglusin, HMGCS1, apoptosis

## Abstract

Venetoclax is used for the priority treatment of elderly patients with acute myeloid leukemia (AML). Resistance or intolerance to venetoclax offsets its clinical benefits in some patients. Combination strategies with other drugs are promising alternatives to overcome the current complications associated with venetoclax use. Hymeglusin, a specific inhibitor of 3-hydroxy-3-methylglutaryl coenzyme A synthase 1 (HMGCS1), regulates the mevalonate pathway, which is vital for AML growth and chemosensitivity. The effects of the combination of venetoclax and hymeglusin on AML were explored in this study. The correlations between HMGCS1 and apoptosis-related genes were analyzed using the Gene Expression Profiling Interactive Analysis 2 and The Cancer Genome Atlas databases. Apoptosis and cell viability were detected in HL-60 and KG-1 cells after treatment with gradient concentrations of venetoclax or hymeglusin. The transcriptomic profiles of HL-60 and KG-1 cells were compared *via* RNA-Seq analysis. The effects of venetoclax and hymeglusin on apoptosis were validated in primary cells. The results showed that HMGCS1 expression was closely associated with apoptosis-related genes based on the data from large clinical databases. B cell lymphoma (BCL)-2 expression was elevated in AML and negatively associated with overall survival. Hymeglusin decreased BCL2 expression levels in HL-60 and KG-1 cells. Venetoclax and hymeglusin inhibited cell viability in both cell lines, but induced apoptosis in HL-60 cells. This discrepancy in sensitivity to hymeglusin may be attributed to the positive increase in the expression levels of HMGCS1 and multiple upregulated pro-leukemia genes in KG-1 cells. Combination treatment with venetoclax and hymeglusin significantly increased the apoptotic rates compared to single-agent treatment in both AML cell lines and primary AML cells. Furthermore, the combination strategy did not result in remarkably enhanced toxicity in normal mononuclear cells. Collectively, hymeglusin enhanced the effects of venetoclax on apoptosis. This combination strategy showed enhanced antileukemic activity with acceptable toxicity in AML.

## Introduction

Acute myeloid leukemia (AML) is a common hematological malignancy in adults, primarily in older patients. The median age at the time of diagnosis was 68 years (1). Intensive chemotherapy is the standard therapy that is beneficial for fitter patients. However, patients with less fit showed poor tolerance to conventional chemotherapy.

As a highly selective antagonist of B-cell lymphoma 2 (BCL2), venetoclax restores apoptosis activation and is recommended for the treatment of elderly or unfit AML patients (2). With the increasing use of venetoclax in AML, acquisition of resistance offsets its clinical benefits. Resistance mechanisms include acquired mutations in BCL2 (3), BAX deletion (4), and alterations in mitochondrial metabolism and epigenetics (5). Therefore, the development of alternative strategies for patients with unfit AML who respond poorly to venetoclax-based therapy is important. Combination strategies using other inhibitors are promising for overcoming venetoclax resistance.

Elderly individuals are characterized by hypercholesterolemia and generally require cholesterol-lowering reagents to prevent cardiovascular disease. A significant increase in *de novo* cholesterol synthesis has been observed in AML (6), which is required for rapid cell growth and is closely associated with chemoinsensitivity (6, 7). Consequently, targeting this metabolic vulnerability by interrupting the increase in protective cholesterol and suppressing its synthesis seems to be an effective strategy for treating AML.

The mevalonate pathway (MVA) is a metabolic pathway for *de novo* cholesterol synthesis. The second step is catalyzed by 3-hydroxy-3-methylglutaryl coenzyme A (HMG-CoA) synthase 1 (HMGCS1), which synthesizes HMG-CoA. HMG-CoA is converted to mevalonate by HMG-CoA reductase (HMGCR), the first committed step in the MVA pathway (8). Hymeglusin was initially described as an antibiotic and was proven to be a specific inhibitor of HMGCS1 (9) by specifically binding to the active site of Cys-129 (10). Previously, we found that HMGCS1 promoted cell growth and increased apoptosis in AML cells (data not shown). However, it is unclear how hymeglusin affects cell apoptosis and its potential clinical applications.

Here, we demonstrated that hymeglusin can reduce the expression of BCL2. Because venetoclax is a specific inhibitor of BCL2, we investigated the combined effects of hymeglusin and venetoclax. The results showed that hymeglusin increased the apoptosis-inducing effect of venetoclax in AML cells, without obvious aggravation of myelosuppression.

## Materials and Methods

### Inhibitors

Hymeglusin (Hym) and Venetoclax (Ven) were purchased from Cayman Chemical (Michigan, USA) and MedChemExpress (Shanghai, China), respectively.

### Cell lines and Culture Conditions

HL-60 and KG-1 cells were purchased from the Procell Life Science & Technology Company (Wuhan, China). They were cultured in Iscove’s modified Dulbecco’s medium (IMDM) (Hyclone, UT, United States) supplemented with 20% fetal bovine serum (Biological Industries Inc., CA, USA) and a 1% antibiotic solution of penicillin and streptomycin (Corning Inc., NY, USA) in a humidified atmosphere containing 5% CO_2_ at 37°C. Hym and Ven treatments were performed at the corresponding times and concentrations.

### Assessment of Apoptosis

A total of 1×10^6^ cells treated with Hym and Ven (at the indicated concentrations), either alone or in combination, were collected after 24–48 h of incubation, washed twice with cold phosphate-buffered saline (PBS), resuspended in 300 μL binding buffer, stained with 5 μL Annexin-V and 10 μL PI (Multisciences Biotech, Hangzhou, China), incubated in the dark for 15 min, and subjected to flow cytometry (Becton Dickinson Bioscience, Oxford, UK) to analyze apoptosis. The IC50 value, or half maximal inhibitory concentration, of each cell line was calculated using the Xiantao web data analysis tool (https://www.xiantao.love). To determine whether the two drugs exerted a synergistic effect, the combination index (CI) was calculated using Compusyn software (ComboSyn Inc., NJ, USA), with the definitions of additive effect (CI = 1), synergism (CI < 1), and antagonism (CI > 1).

### Cytotoxicity Assay

To evaluate cell response to different drugs, cell viability was determined using the Cell Counting Kit-8 (CCK-8) (Dojin Laboratories, Kumamoto, Japan) after treatment. HL-60 and KG-1 cells (5×10^4^/ml) were seeded in 96-well culture plates and incubated with Hym and Ven at the indicated concentrations alone or in combination. After certain time, 10 μL CCK-8 solution was added to each well for a 3 h culture at 37°C. Absorbance was measured using a spectrophotometer (BioTek Instruments, US) at a wavelength of 450 nm. The IC50 value of each cell line was calculated using the Xiantao web data analysis tool (https://www.xiantao.love). The confidence interval (CI) was also calculated to determine synergism between the two drugs.

### Quantitative RT-PCR

HL-60 and KG-1 cells were treated with Hym at concentrations of 8 μM and 16 μM, respectively. After 24 h, cells were collected. RNA was isolated from 1× 10^6^ cells using TRIzol reagent (TaKaRa, Kusatsu, Japan). Total RNA (1000 ng) was reverse-transcribed into first-strand cDNA using the Prime ScriptTM RT Reagent Kit (TaKaRa, Japan, Cat#RR047A). cDNA was amplified according to the manufacturer’s instructions using a transcription kit (TaKaRa, Japan, Cat#RR047A). Primers for real-time PCR were obtained from the Tsingke Biotechnology Company (Beijing, China). The primer sequences used are listed in **Supplementary Table 1**. Reactions were performed on an Applied Biosystems Prism machine using an ABI StepOnePlus (Applied Biosystems, Foster City, CA, USA). Transcript levels of the genes of interest were normalized to GAPDH transcript levels.

### Western Blotting Analysis

Equal amounts of protein were separated *via* 10% sodium dodecyl sulfate-polyacrylamide gel electrophoresis and transferred electrophoretically to polyvinylidene fluoride membranes (Millipore, Billerica, MA, USA). The membranes were incubated with TBS-T containing 5% milk for an hour at room temperature and then with primary antibodies overnight at 4°C. After further incubation with secondary antibodies, protein bands were detected using a Chei DocTMMP imaging system (BioRad, CA, USA). Primary antibodies for Caspase-3, BCL2, and BAX were purchased from Wanleibio Company (Shenyang, China). PUMA antibody was purchased from ABclonal (Wuhan, China). SREBP2 antibody was purchased from Affinity Biosciences (Jiangsu, China). Anti-HMGCS1 and anti-GAPDH antibodies were purchased from Abcam (Boston, MA, USA) and Cell Signaling Technology (Danvers, Massachusetts, USA), respectively.

### Isolation of Mononuclear Cells (MNCs)

Bone marrow samples (approximately 1 mL) from bone marrow samples from *de novo* patients with AML or peripheral blood samples from healthy donors were collected. The samples were diluted 1:1 with sterile PBS. The cell suspension was then added to the top of a 2 mL lymphocyte separation solution (Ficoll-Paque). The mixture was centrifuged at 2000 RPM for 20 min. The MNC layer was aspirated into a new tube and washed twice with PBS. Finally, MNCs were resuspended in complete cell culture medium and seeded in 24-well plates for venetoclax (0.1 μM) with or without hymeglusin (16 μM) for 24 h.

### RNA-Seq Analysis

Approximately 1×10^7^ HL-60 and KG-1 cells (both including control and 24 h hymeglusin treatment groups) were collected and suspended in Trizol (TaKaRa, Kusatsu, Japan). The cells were then sent to Tsingke Biotechnology (Beijing, China) for RNA isolation and sequencing.

### Gene Expression and Survival Prognosis Analysis

The “Expression analysis-Box Plots” module of the Gene Expression Profiling Interactive Analysis 2 (GEPIA2) web server (http://gepia2.cancer-pku.cn/) was used to obtain box plots of the gene expression differences between AML and normal individuals. The overall survival analysis of genes is presented in the “Survival” tab. Log-rank P-values and hazard ratios (HR) with 95% CIs were also calculated. The dataset in the Gene Expression Omnibus (GEO) Profiles (Accession: GDS3057, Series: GSE9476) was analyzed. The expression profiles of apoptosis-related genes from the peripheral blood mononuclear cells of 19 Patients with AML and 10 healthy donors were compared.

### Correlation Analysis of Gene Expression

We used the Xiantao web (https://www.xiantao.love) to assess the relationship between the expression of the two genes in AML. Xiantao is a web server for analyzing RNA sequencing data from TCGA projects. A total of 151 patients with AML were included in TCGA database. The patients were evenly divided based on HMGCS1 expression levels. Spearman’s correlation test was used to assess correlations.

### Gene Enrichment Analysis

The lists of genes and transcripts with significant changes in DE were exported from RNA-seq and further utilized for Kyoto Encyclopedia of Genes and Genomes (KEGG) and Gene Ontology (GO) enrichment analysis using the “KEGG/GO enrichment visualization” tool of XianTao.

### Statistical Analysis

Data are represented as the mean ± standard deviation of three replicate experiments. Differences between groups were analyzed using Student’s *t*-test or one-way analysis of variance, as appropriate. Statistical analyses were performed using the GraphPad Prism software (version 8.0). Statistical significance was set at P < 0.05. *P < 0.05, **P < 0.01, ***P < 0.001, ****P < 0.0001.

### Ethics Statement

This study conformed to the guidelines and was approved by the Ethics Committee of Xiangya Hospital, Central South University. Signed informed consent was obtained from all participants.

## Results

### HMGCS1 Expression Is Correlated With the Expression of Genes Related to Apoptosis

**Figure 1:**
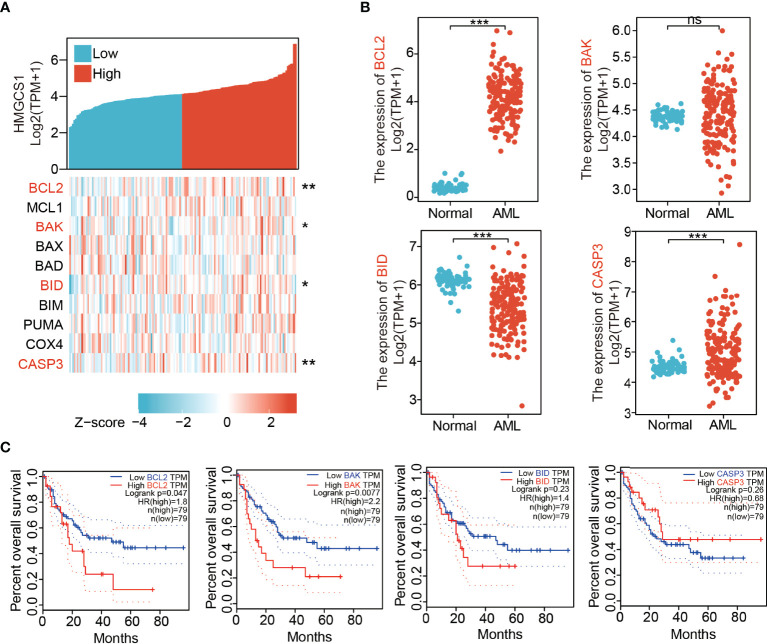
Expression levels of 3-hydroxy-3-methylglutaryl-CoA synthase 1 (HMGCS1) correlated with those of apoptosis-related genes. **(A)** The correlation between HMGCS1 level and key apoptosis related genes by analyzing dataset in Xiantao. The genes included BCL2, MCL1, BAK, BAX, BAD, BID, BIM, PUMA, COX4, and CASP3. **(B)** The expression level of BCL2, BAK, BID, and CASP3 between patients with AML and normal controls utilizing the GTEx and TCGA databases. **(C)** The association of BCL2, BAK, BID, and CASP3 level with overall survival. *P<0.05, **P<0.01, ***P<0.001. ns, not significant.

We analyzed the correlation between HMGCS1 expression and key genes (BCL2, MCL1, BAK, BAX, BAD, BID, BIM, PUMA, COX4, and CASP3) that regulate apoptosis, using the Xiantao dataset (**Figure 1A**). HMGCS1 expression was significantly correlated with BCL2, BAK, BID, and CASP3 expression (**Figure 1A**). We then compared the expression of apoptosis-related genes between AML and normal patients in the GTEx and TCGA databases. The results indicated that the levels of BCL2, CASP3 (**Figure 1B**) and MCL1 (**Supplementary Figure 1C**) were significantly higher in AML. In contrast, the levels of BID (**Figure 1B**), BIM, PUMA (**Supplementary Figure 1A**), BAD, and COX4 (**Supplementary Figure 1C**) were lower in AML. Differences in the expression of BAK (**Figure 1B**) and BAX (**Supplementary Figure 1C**) were not significant. Further survival analysis using the GEPIA2 database showed that BCL2, BAK (**Figure 1C**), BIM, and PUMA (**Supplementary Figure 1B**) were negatively associated with overall survival (OS) in AML. The effect of BID, CASP3 (**Figure 1C**), MCL1, BAX, BAD, and COX4 (**Supplementary Figure 1D**) on OS was minimal. The expression of apoptosis-related genes in healthy donors and patients with AML was further compared based on the GEO profile dataset. The results showed that HMGCS1, BCL2, and BAX were elevated in patients with AML, whereas BID and BIM were downregulated in AML. MCL1, BAK, BAD, COX4, and CASP3 were not significantly different between patients with AML and healthy donors (**Supplementary Figure 1E**). BCL2, BAK, and BID, three of the four genes that were significantly correlated with HMGCS1 expression, showed similar alterations in both TCGA and GEO datasets. These data revealed that HMGCS1 expression was correlated with some apoptosis-related genes, especially BCL2, which significantly affects OS in AML.

### Hymeglusin Reduces BCL2 Expression in AML Cells

**Figure 2:**
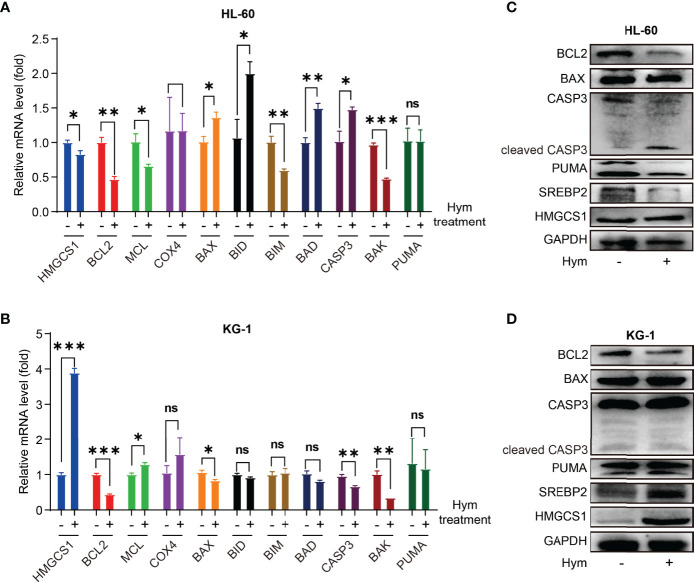
Impact of hymeglusin on the expression levels of apoptosis-related genes. **(A, B)** The RNA level alterations of important genes involved in apoptosis after hymeglusin treatment in HL-60 **(A)** and KG-1 cells **(B)**. **(C, D)** The protein levels of key apoptosis related genes (BCL2, BAX, PUMA, CASP3 and cleaved CASP3), and SREBP2 after hymeglusin treatment in HL-60 **(C)** and KG-1 cells **(D)**. The treatment concentrations were 8 μM and 16 μM for HL-60 and KG-1 cells, respectively. *P<0.05,**P<0.01,***P<0.001. ns, not significant

To verify these results, we treated HL-60 and KG-1 cells with hymeglusin, a specific inhibitor of HMGCS1. The transcription and translation levels of genes associated with apoptosis (BCL2, MCL1, COX4, BAX, BID, BIM, BAD, CASP3, BAK, and PUMA) were determined. Levels of pro-apoptotic genes, such as BAX, BID, BAD, and CASP3, were elevated after hymeglusin treatment in HL-60 cells. However, in KG-1 cells, the apoptosis-promoting genes COX4, BAX, BID, BIM, BAD, CASP3, BAK, and PUMA were not upregulated after hymeglusin treatment. Although hymeglusin exerted different effects on genes associated with apoptosis in HL-60 and KG-1 cell lines, it reduced the RNA (**Figures 2A, B**) and protein levels (**Figures 2C, D**) of BCL2. Surprisingly, HMGCS1 levels were elevated in KG-1 cells (**Figures 2B, D**) but not in HL-60 cells (**Figures 2A, C**). Sterol regulatory element binding protein 2 (SREBP2) is the main transcriptional factor targeting HMGCS1 (11, 12). We tested whether its expression was affected by hymeglusin. Consistent with the changes in HMGCS1 levels, SREBP2 levels increased profoundly in KG-1 but not in HL-60 cells after hymeglusin treatment (**Figures 2C, D**).

### Venetoclax and Hymeglusin Induce the Loss of Cell Viability in AML Cells

**Figure 3:**
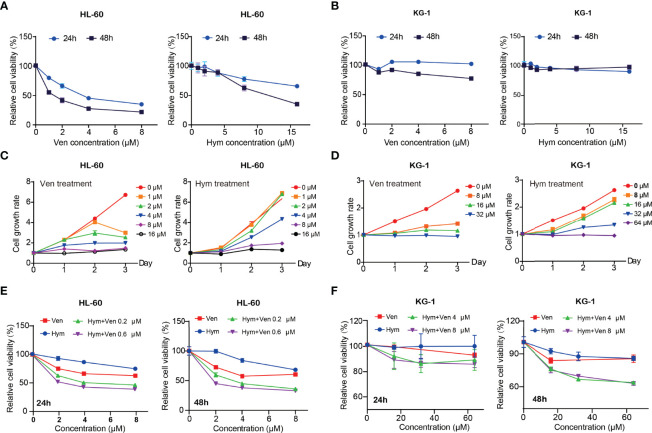
Venetoclax and hymeglusin inhibit the cell viability in acute myeloid leukemia (AML) cells. **(A, B)** The relative cell viability curve after gradient concentration of venetoclax (0–8 μM) and hymeglusin (0-16 μM) treatment for 24 to 48 hours in HL-60 **(A)** and KG-1 cells **(B)**. **(C)** Cell growth rates after venetoclax (0–16 μM) and hymeglusin (0–16 μM) treatment for 24–72 h in HL-60 cells. **(D)** Cell growth rates after venetoclax (0-32 μM) and hymeglusin (0–64 μM) treatment for 24 to 72 hours in KG-1 cells. **(E)** The relative cell viability alterations after venetoclax (0.2 μM) and hymeglusin (0.6 μM) treatment for 24 to 48 hours in HL-60 cells. **(F)** The relative cell viability alterations after venetoclax (4 μM) and hymeglusin (8 μM) treatment for 24 to 48 hours in KG-1 cells.

Both venetoclax and hymeglusin inhibited AML cell viability (**Figures 3A–D**). In HL-60 cells, the IC50s of venetoclax were 4.06 μM (48 h) and 0.51 μM (72 h) (**Supplementary Figure 2A**); the IC50s of hymeglusin were 7.65 μM (48h) and 5.96 μM (72 h) (**Supplementary Figure 2B**). As for KG-1 cells, the IC50s of venetoclax were over 32 μM (48 h) and 10.73 μM (72 h) (**Supplementary Figure 2C**); the IC50s of hymeglusin were 61.64 μM (48h) and 38.95 μM (72h) (**Supplementary Figure 2D**). Collectively, HL-60 cells were more sensitive to venetoclax and hymeglusin than KG-1 cells. Hymeglusin sensitized HL-60 and KG-1 cells to venetoclax. As hymeglusin decreased BCL2 expression, venetoclax could inhibit the activity of BCL2; thus, a combination strategy of both inhibitors was considered. Their co-exposure resulted in enhanced inhibitory effects on AML cell viability (**Figures 3E, F**). Synergistic effects were observed in HL-60 cells, as indicated by CI values < 1 (range 0.32 to 0.65), regardless of drug concentrations and treatment time points.

### Venetoclax and Hymeglusin Induce Apoptosis in AML Cells

**Figure 4:**
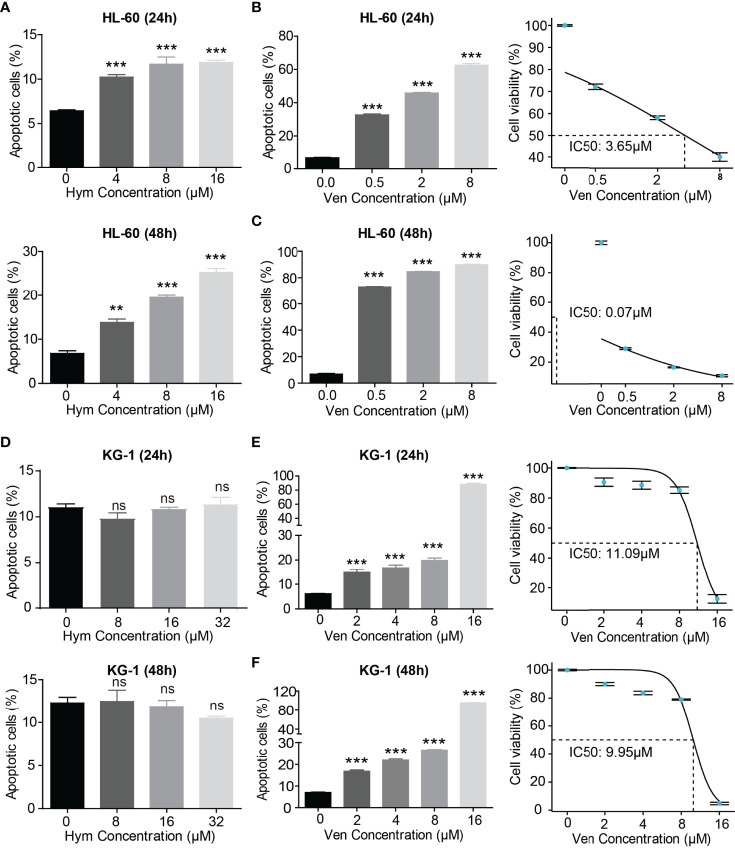
Venetoclax and hymeglusin affect the apoptosis of AML cells. **(A)** The apoptotic rate after gradient concentration of hymeglusin (0–16 μM) treatment for 24 to 48 hours in HL-60 cells. **(B, C)** The apoptotic rate after gradient concentration of venetoclax (0–8 μM) treatment for 24 **(B)** to 48 h **(C)** in HL-60 cells. The cell viability curve indicating IC50 was shown in the right panel. **(D)** The apoptotic rate after gradient concentration of hymeglusin (0–32 μM) treatment for 24–48 h in KG-1 cells. **(E, F)** The apoptotic rate after gradient concentration of venetoclax (0–16 μM) treatment for 24 **(E)** to 48 h **(F)** in KG-1 cells. The right panel shows the cell viability curve indicating IC50. **P<0.01, ***P<0.001. ns, not significant.

**Figure 5:**
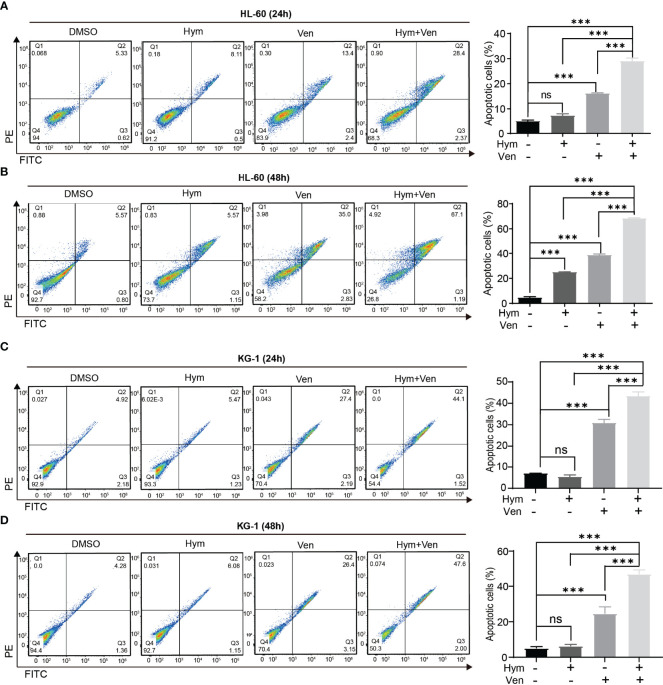
Combined treatment with venetoclax and hymeglusin promotes the apoptosis of AML cells. **(A, B)** The percentage of apoptotic cells after venetoclax (0.1 μM) and hymeglusin (16 μM) treatment for 24 **(A)** to 48 h **(B)** in HL-60 cells. The left panel shows the flow cytometry scatter plot. **(C, D)** The percentage of apoptotic cells after venetoclax (10 μM), hymeglusin (16 μM) or both treatment for 24 **(C)** to 48 h **(D)** in KG-1 cells. The flow cytometry scatter plots were shown the left part. ***P<0.001. ns, not significant.

The percentage of apoptotic cells elevated with increasing concentrations of hymeglusin (**Figure 4A**; **Supplementary Figures 3A, B**) and venetoclax (**Figures 4B, C**; **Supplementary Figures 3C, D**) in HL-60 cells. The IC50 of venetoclax was 3.65 μM (24h) and 0.07 μM (48h) (**Figures 4B, C**), whereas the IC50 of hymeglusin was over 16 μM. Similarly, venetoclax induced apoptosis in KG-1 cells (**Figures 4E, F**; **Supplementary Figures 4C, D**). The IC50 values of venetoclax were 11.09 μM (24 h) and 9.95 μM (48 h) (**Figures 4E, F**). However, hymeglusin had minimal effects on apoptosis in KG-1 cells (**Figure 4D**; **Supplementary Figures 4A, B**). However, the combination of hymeglusin and venetoclax markedly improved apoptosis in both HL-60 (**Figures 5A, B**) and KG-1 cells (**Figures 5C, D**) compared to monotherapy. Furthermore, the combination strategy showed strong synergistic effects in HL-60 cells (CI range 0.24 to 0.35).

### Differences in Transcriptomic Profiles Between HL-60 and KG-1 Cells

**Figure 6:**
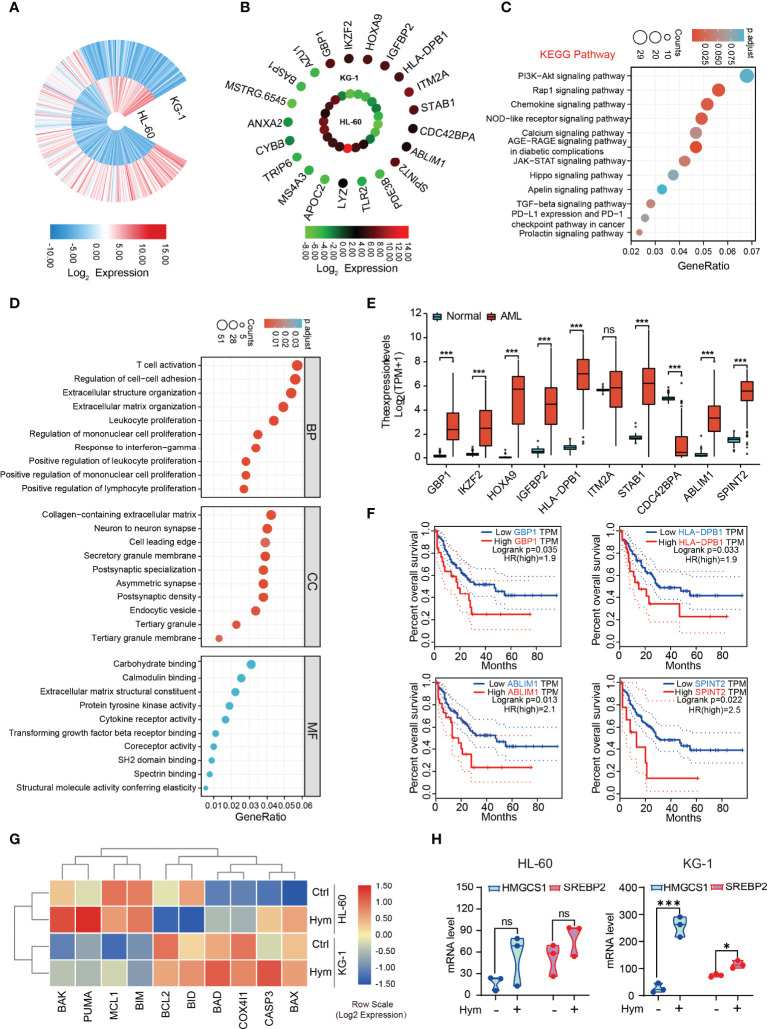
Comparison of transcriptomic profiles between HL-60 and KG-1 cells. **(A)** The gene expression differences between HL-60 and KG-1 cells resulted from analysis of RNA-seq. **(B)** The top ranked 20 genes with the highest differential expression level between HL-60 and KG-1 cells. **(C, D)** Functional enrichment analyses by KEGG **(C)** and GO **(D)**. The top ten terms in each analysis were listed. The color of the dot represents adjusted p-value. Size of the dot represents the number of genes associated with each term. **(E)** The expression discrepancy of the top 10 differentially expressed genes with high level in KG-1 cells between AML and normal controls. **(F)** The influence of GBP1, HLA-DBP1, ABLIM1, and SPINT2 expression on OS in AML. **(G)** The heatmap of apoptosis related genes expressions after hymeglusin treatment, revealed by RNA-seq. **(H)** The alterations of HMGCS1 and SREBP2 expressions after hymeglusin treatment, revealed by RNA-seq. *P<0.05, ***P<0.001. ns, not significant.

We explored why HL-60 and KG-1 cells showed different sensitivities to venetoclax and hymeglusin. By analyzing RNA-Seq data, we found that there was a distinct gene expression pattern between these two cell lines (**Figure 6A**). The top 10 genes with the highest expression in KG-1 cells were GBP1, IKZF2, HOXA9, IGFBP2, HLA-DBP1, ITM2A, STAB1, CDC42BPA, ABLIM1, and SPINT2. The top 10 genes with the highest expression in HL-60 cells were AZU1, BASP1, MSTRG.6545, ANXA2, CYBB, TRIP6, MS4A3, APOC2, LYZ, TLR2, and PDE3B (**Figure 6B**). KEGG pathway analysis revealed enrichment of the Rap1 signaling pathway, chemokine signaling, and NOD receptor signaling pathways (**Figure 6C**). GO analysis showed that the three main terms enriched in the biological process (BP) ontology were T cell activation, regulation of cell adhesion, and organization of extracellular structures. For the Cellular Component (CC) ontology, the three most enriched terms included collagen-containing extracellular matrix, neuron-to-neuron synapse, and cell leading edge. For the molecular function (MF) ontology, the top three terms were carbohydrate binding, calmodulin binding, and structural components of the extracellular matrix (**Figure 6D**). The expression of many of the highest-ranked genes in KG-1 cells was significantly higher in AML than in normal controls (**Figure 6E**). Among them, GBP1, HLA-DBP1, ABLIM1, and SPINT2 were negatively correlated with OS in AML (**Figure 6F**). To further investigate the effect of hymeglusin on AML cells, RNA-Seq data of HL-60 and KG-1 cells with or without hymeglusin treatment were compared (**Figures 6G, H**). The results showed that after hymeglusin treatment, the expression of apoptosis-related genes, such as BCL2, was altered (**Figure 6G**). Consistent with the above data, HMGCS1 and SREBP2 levels were significantly elevated in KG-1 cells but not in HL-60 cells after hymeglusin treatment (**Figure 6H**).

### Hymeglusin Improves the Efficacy of Venetoclax in Patients With AML

**Figure 7:**
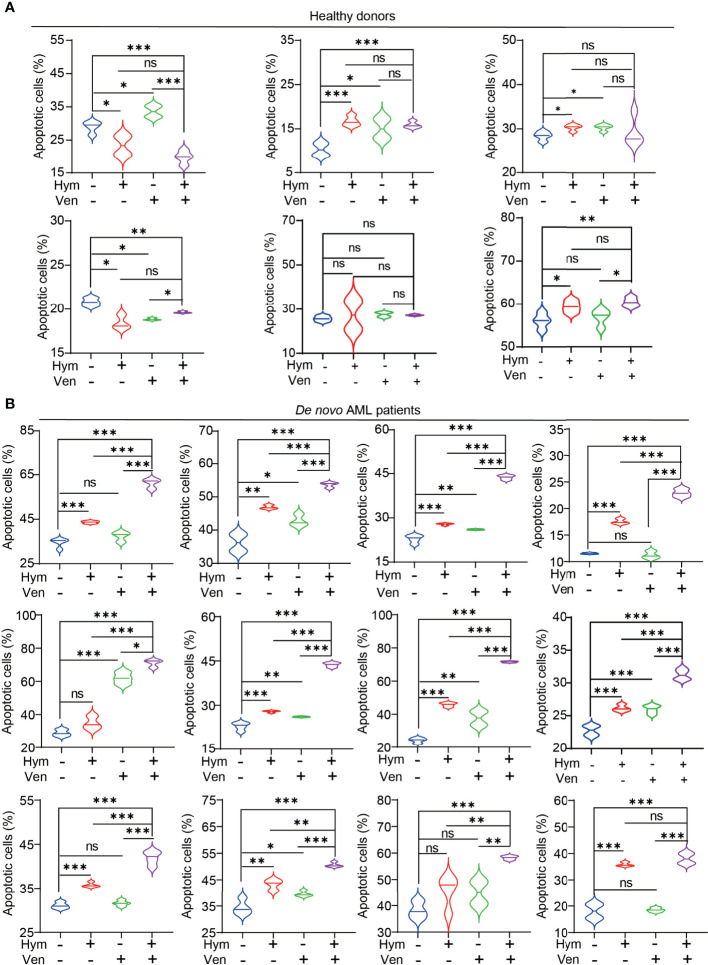
Effects of hymeglusin and venetoclax on the apoptosis of primary cells. **(A, B)** The apoptotic rates after treatment of venetoclax (0.1 μM), hymeglusin (16 μM), or both for 24 hours in primary cells from healthy donors **(A)** or *de novo* patients with AML **(B)** *P<0.05, ***P<0.001.

Finally, we tested the effects of venetoclax and hymeglusin on the apoptosis of primary cells. Consistently, the effects of hymeglusin in healthy donors were less obvious than those in patients with *de novo* AML. The addition of hymeglusin did not result in an obvious increase in apoptotic rates compared with monotherapy with venetoclax (**Figure 7A**; **Supplementary Figure 5A**). However, hymeglusin significantly increased venetoclax-induced apoptotic rates in primary AML cells (**Figure 7B**; **Supplementary Figure 5B**).

## Discussion

Venetoclax-based combinations have been investigated in AML, particularly in subgroups of patients with unfit and relapsed/refractory AML. Combined agents with preclinical evidence in AML models include hypomethylating agents (HMA), low-dose cytarabine (LDAC), intensive chemotherapy, IDH1/2 inhibitors, and FLT3 inhibitors (13). Our results highlight the remarkable pro-apoptotic effects of hymeglusin and venetoclax in AML cells. Mechanistically, venetoclax inhibited BCL2 activity, while hymeglusin could reduce the expression of BCL2. This combination strategy is encouraging because it offers several advantages. First, our previous data demonstrated HMGCS1 overexpression in patients with AML (data not shown). Addition of hymeglusin decreased the oncogenic function of HMGCS1. In addition, a certain proportion of the elderly patients had concurrent cardiovascular complications and were prescribed cholesterol-lowering agents. Hymeglusin was thought to reduce cholesterol levels. Therefore, the application of hymeglusin can not only promote venetoclax sensitivity by elevating leukemia cell apoptosis but also control serum cholesterol levels. Furthermore, the recommended dose of venetoclax for patients with AML is 400 mg per day (14). However, in our clinical practice, some patients were intolerable due to severe marrow suppression. Reducing the dose to 100 mg per day resulted in better tolerance. Our study showed that similar effects were achieved with a lower concentration of venetoclax combined with hymeglusin than with venetoclax alone. Thus, this combination strategy may overcome the severe adverse events associated with myelosuppression.

Statins are specific inhibitors of HMGCR and are used clinically to control cholesterol levels. Statins can promote apoptosis of AML cells (15). Lee et al. showed that simvastatin promoted venetoclax cytotoxicity in lymphoma and AML cells. HMGCR blockage suppresses geranylgeranylation, which upregulates the pro-apoptotic protein p53 and upregulates the modulator of apoptosis (PUMA) (16). However, in other reports, the results were unsatisfactory, with an increase in the expression of HMGCR and HMGCS1 after statin treatment, which was attributed to the classic feedback response. This feedback could offset the anti-leukemic effects of statins (17). HMGCS1 catalyzes the enzymatic step prior to HMGCR, which is crucial for controlling the influx of the MVA pathway. Therefore, HMGCS1 is a promising target for inhibiting MVA pathway activity. A comparison of the impacts on apoptosis suppression after inhibiting HMGCS1 or HMGCR required further exploration.

HL-60 and KG-1 cells showed variable responses to hymeglusin and venetoclax. This cooperation appeared to be more effective in HL-60 cells than in KG-1 cells. Hymeglusin promoted expressions of pro-apoptotic genes in HL-60 cells, but not in KG-1 cells. This was consistent with the apoptosis assay indicating that hymeglusin induced apoptosis in HL-60 cells, but no significant apoptotic effect on KG-1 cells. After hymeglusin treatment, the expression of HMGCS1 and SREBP2 was obviously elevated in KG-1 cells, but not in HL-60 cells (**Figures 2**, **6H**). This feedback increase in HMGCS1 expression may be another reason why KG-1 was not as sensitive as HL-60 cells to hymeglusin. Dipyridamole blocks the restorative feedback loop after statin treatment (17). The combination of hymeglusin and dipyridamole may be a potential strategy to enhance the antitumor potency of hymeglusin. However, it was encouraging to find that KG-1 cells responded well to the combined treatment with hymeglusin and venetoclax. Taken together, the hymeglusin and venetoclax regimen is a promising treatment for AML.

To further investigate the factors underlying the difference in drug sensitivity, we compared the transcriptomic profiles of HL-60 and KG-1 cells. Six of the ten genes that were highly expressed in KG-1 cells were reported to be closely associated with AML. For example, GBP1 expression is lower in patients with leukemia and yields a survival benefit for these patients (18). IKZF2 is highly expressed in stem cells from myeloid leukemia and is required for leukemogenesis by blocking their differentiation (19). HOXA9 is an oncogenic transcription factor that promotes leukemogenesis. HOXA9 overexpression is prominent in AML and is associated with a poor prognosis (20, 21). Overexpression of IGFBP2 not only promotes infiltration of AML cells into peripheral organs and tissues (22) but also confers chemoresistance (23). STAB1 is a prognostic factor for poor outcomes in cytogenetically normal patients with AML. Its high expression reduces the sensitivity of venetoclax (24). The specific roles of the remaining four genes in AML require further investigation. Overexpression of genes that promote leukemia in KG-1 cells may be responsible for the relatively inferior response of these cells to hymeglusin and venetoclax treatment.

A concern regarding the combined regimen was whether it would exacerbate any single-agent toxicity. In primary cells, the combinatorial strategy with hymeglusin and venetoclax showed significantly enhanced antileukemia activity in newly diagnosed patients with AML, with acceptable toxicities in normal PBMC. This indicated the efficacy and safety of this combination to some extent.

A limitation of this study was that there was only *in vitro* evidence supporting the superior antileukemic effect of the cotreatment with hymeglusin and venetoclax. Further preclinical investigation in animal models and clinical evaluation in patients with AML are warranted to validate these results, which will be the focus of our future research.

## Data Availability Statement

The datasets presented in this study can be found in online repositories. The names of the repository/repositories and accession number(s) can be found below: ENA (https://www.ebi.ac.uk/ena/browser/home), ERR9237191, ERR9134927, ERR9134928, ERR9134929, ERR9134930, ERR9237356. The accession numbers of RNA-Seq data about HL-60 and KG-1 treated by hymeglusin are: ERR9669119, ERR9669305, ERR9669151, ERR9679661, ERR9669491, ERR9669540.

## Ethics Statement

The studies involving human participants were reviewed and approved by Ethics Committee of Xiangya Hospital, Central South University. The patients/participants provided their written informed consent to participate in this study.

## Author Contributions

LZ designed the project; CZ and ZW performed the experiments; SY and HL analyzed the data; CZ and LZ wrote the manuscript; All authors have read and approved the final submitted manuscript.

## Funding

This project was supported by the National Natural Science Foundation of China (Grant No. 81800174) and the Natural Science Foundation of Hunan Province, China (Grant No. 2021JJ41056 and 2020JJ5927).

## Conflict of Interest

The authors declare that the research was conducted in the absence of any commercial or financial relationships that could be construed as a potential conflict of interest.

## Publisher’s Note

All claims expressed in this article are solely those of the authors and do not necessarily represent those of their affiliated organizations, or those of the publisher, the editors and the reviewers. Any product that may be evaluated in this article, or claim that may be made by its manufacturer, is not guaranteed or endorsed by the publisher.
