# Supplementary material for: Hymeglusin Enhances the Pro-Apoptotic Effects of Venetoclax in Acute Myeloid Leukemia
Source: Front Oncol. 2022 Jun 29;12:864430. doi: 10.3389/fonc.2022.864430 (PMC9277771; doi:10.3389/fonc.2022.864430)

**Supplementary tables**

**Supplementary table1 Primers for quantitative RT-PCR**

| **Genes** | **Forward primer sequence** | **Reverse primer sequence** |
| --- | --- | --- |
| HMGCS1 | CTTCAGGTTCTGCTGCTGTG | CAGAAGAACTTACGCTCGGC |
| GAPDH | CTTTGTCAAGCTCATTTCCTGG | TCTTCCTCTTGTGCTCTTGC |
| BCL2 | GTCTTCGCTGCGGAGATCAT | CATTCCGATATACGCTGGGAC |
| MCL | TGCTTCGGAAACTGGACATCA | TAGCCACAAAGGCACCAAAAG |
| COX4 | CGACAGACCTTACAAAGACATCC | GAAGAATAAGATCCCAGCCGT |
| BAX | CCCGAGAGGTCTTTTTCCGAG | CCAGCCCATGATGGTTCTGAT |
| BID | CTGGACATTACTGGGGGCAG | CTCGATAGCCCCTTGGTGTC |
| BIM | CATATAACCCCGTCAACGCAG | GCAGCCGCCACAAACATAC |
| BAD | GGGCACAGCAACGCAGATG | TGGGAACGGGTGGAGTTTCG |
| CASP3 | CATGGAAGCGAATCAATGGACT | CTGTACCAGACCGAGATGTCA |
| BAK | GTTTTCCGCAGCTACGTTTTT | GCAGAGGTAAGGTGACCATCTC |
| PUMA | GCCAGATTTGTGAGACAAGAGG | CAGGCACCTAATTGGGCTC |

**Supplementary Figure Captions**

**Supplementary Figure 1. Expression levels of apoptosis-related genes in acute myeloid leukemia (AML) and its impacts on overall survival (OS).**

(**A**) The gene expression of BIM and PUMA between AML and controls. (**B**) The association of BIM and PUMA expression with OS in AML. (**C**) The gene expression of MCL1, BAX, BAD and COX4 between AML and controls. (**D**) The association of MCL1, BAX, BAD and COX4 expression with OS in AML. (**E**) The comparison of apoptosis related gene expressions between normal donors (N) and patients with AML (P) from GEO profiles.

**Supplementary Figure 2. Cell viability curve after venetoclax and hymeglusin treatment in AML cells.**

(**A–B**) Relative cell viability after gradient concentrations of venetoclax (0–16 μM) (**A**) and hymeglusin (0–16 μM) (**B**) treatment for 24–72 h in HL-60 cells. (**C–D**) Relative cell viability after gradient concentration of venetoclax (0–32 μM) (**C**) and hymeglusin (0–64 μM) (**D**) treatment for 24–72 h in KG-1 cells.

**Supplementary Figure 3. Pro-apoptotic effects of venetoclax and hymeglusin in HL-60 cells.**

(**A–B**) Representative flow cytometry scatter plots showing apoptotic rates after treatment with a gradient concentration of hymeglusin (0–16 μM) for 24 (**A**) to 48 h (**B**). (**C–D**) Representative flow cytometry scatter plots showing apoptotic rates after treatment with a gradient concentration of venetoclax (0–8 μM) for 24 (**C**) to 48 h (**D**).

**Supplementary Figure 4. Impacts of venetoclax and hymeglusin on the apoptosis of KG-1 cells.**

(**A–B**) Representative flow cytometry scatter plots showing apoptotic rates after treatment with a gradient concentration of hymeglusin (0–32 μM) for 24 (**A**) to 48 h (**B**). (**C–D**) Representative flow cytometry scatter plots showing apoptotic rates after treatment with a gradient concentration of venetoclax (0–16 μM) for 24 (**C**) to 48 h (**D**).

**Supplementary Figure 5. Venetoclax and hymeglusin affect the apoptosis of primary cells.**

(**A–B**) Representative flow cytometry scatter plots showing the apoptotic rates after treatment with venetoclax (0.1 μM), hymeglusin (16 μM), or both for 24 h in primary healthy donors (**A**) and *de novo* patients with AML (**B**).

**Supplementary Figures**

**Figure 1**

**
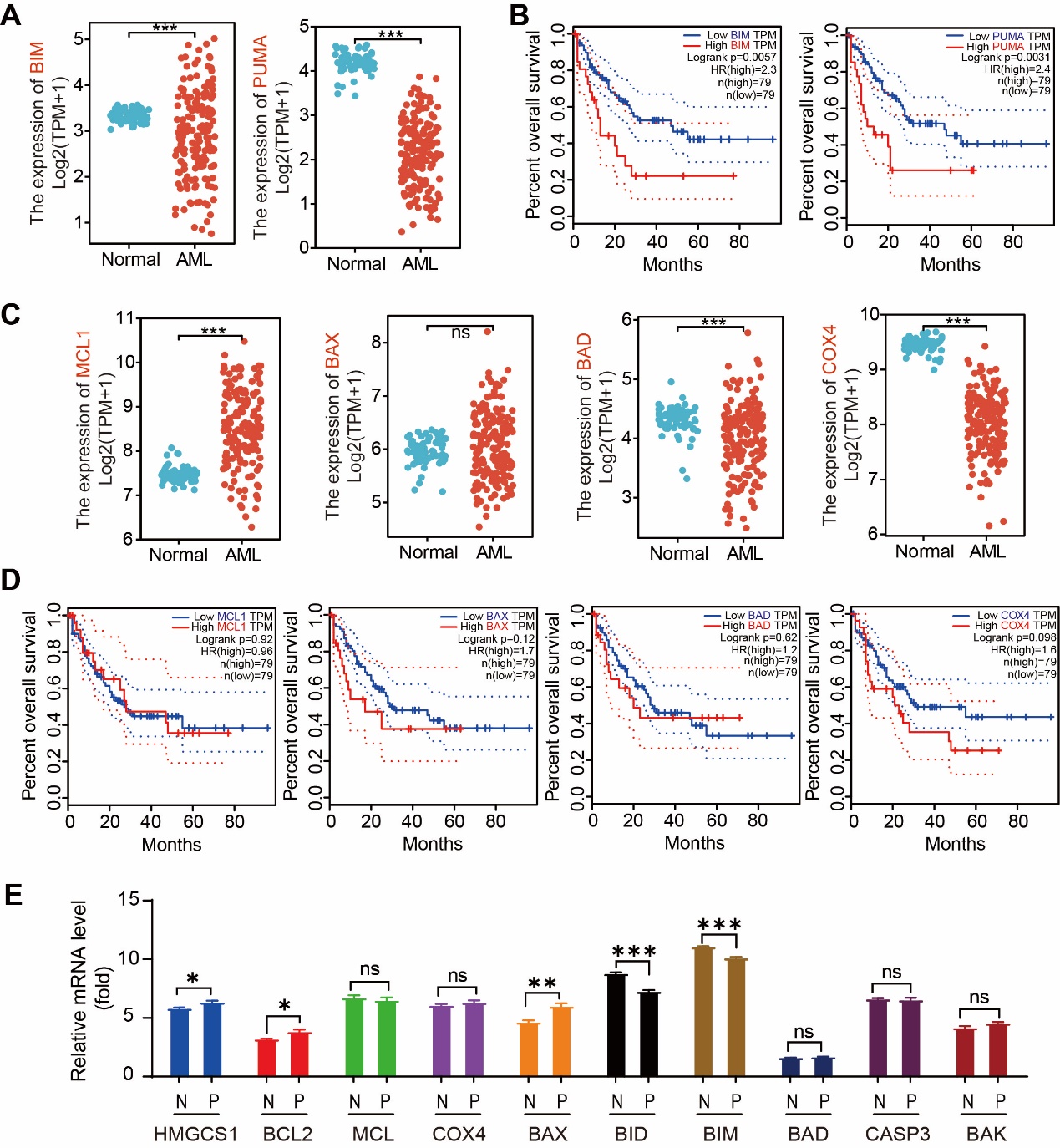
**

**­**

**Figure 2**

**
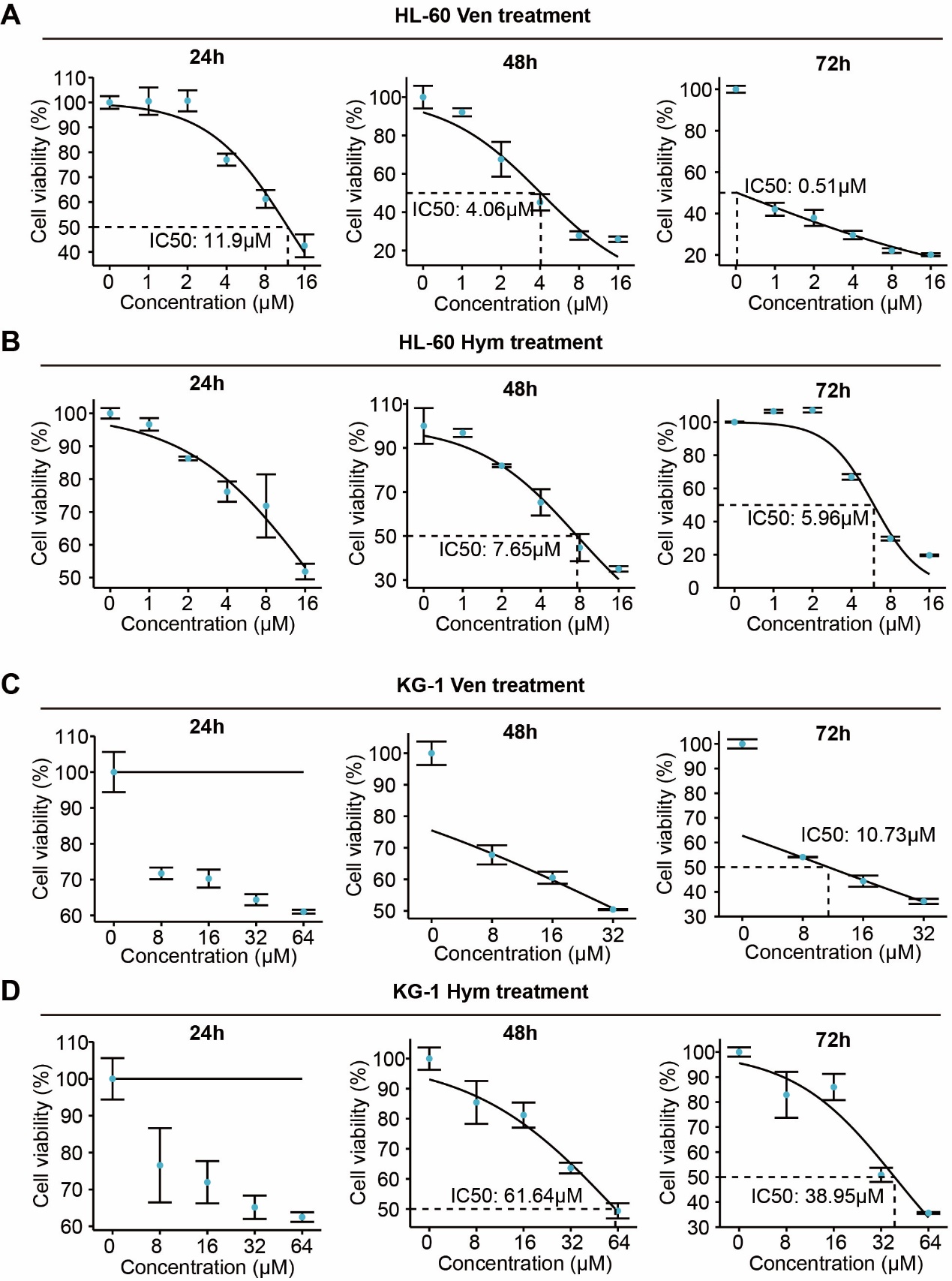
**

**Figure 3**

**
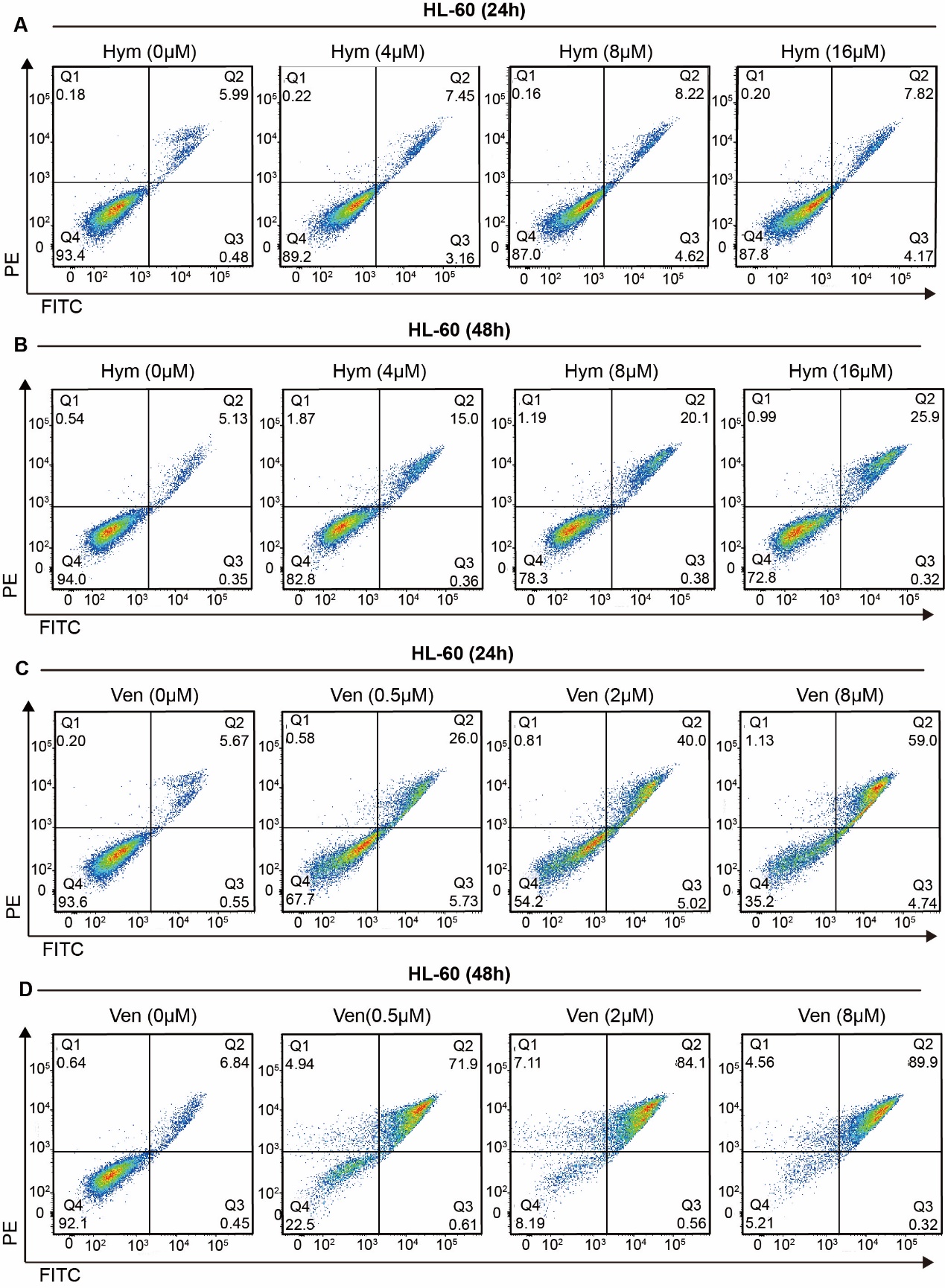
**

**Figure 4**


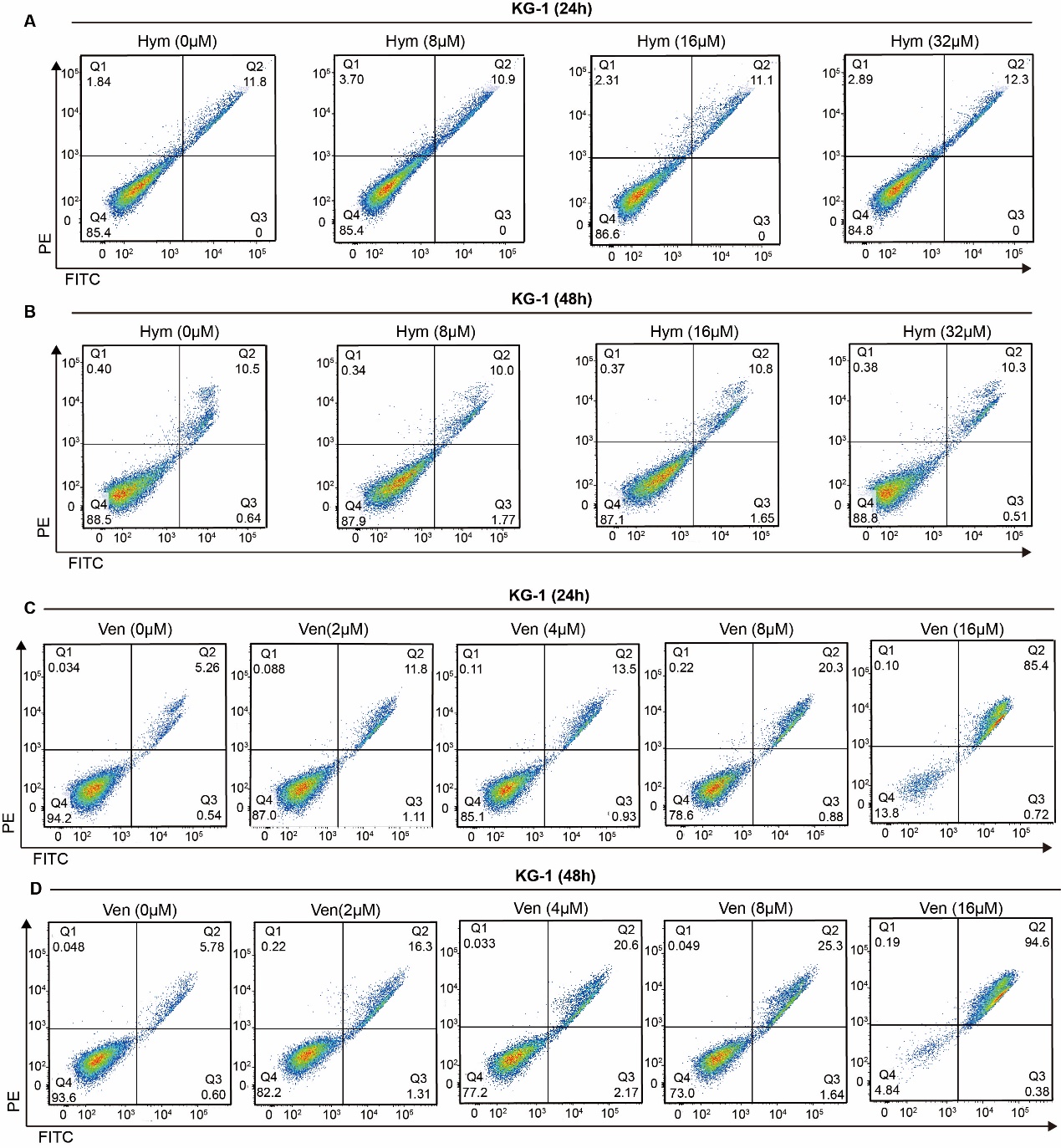


**Figure 5**


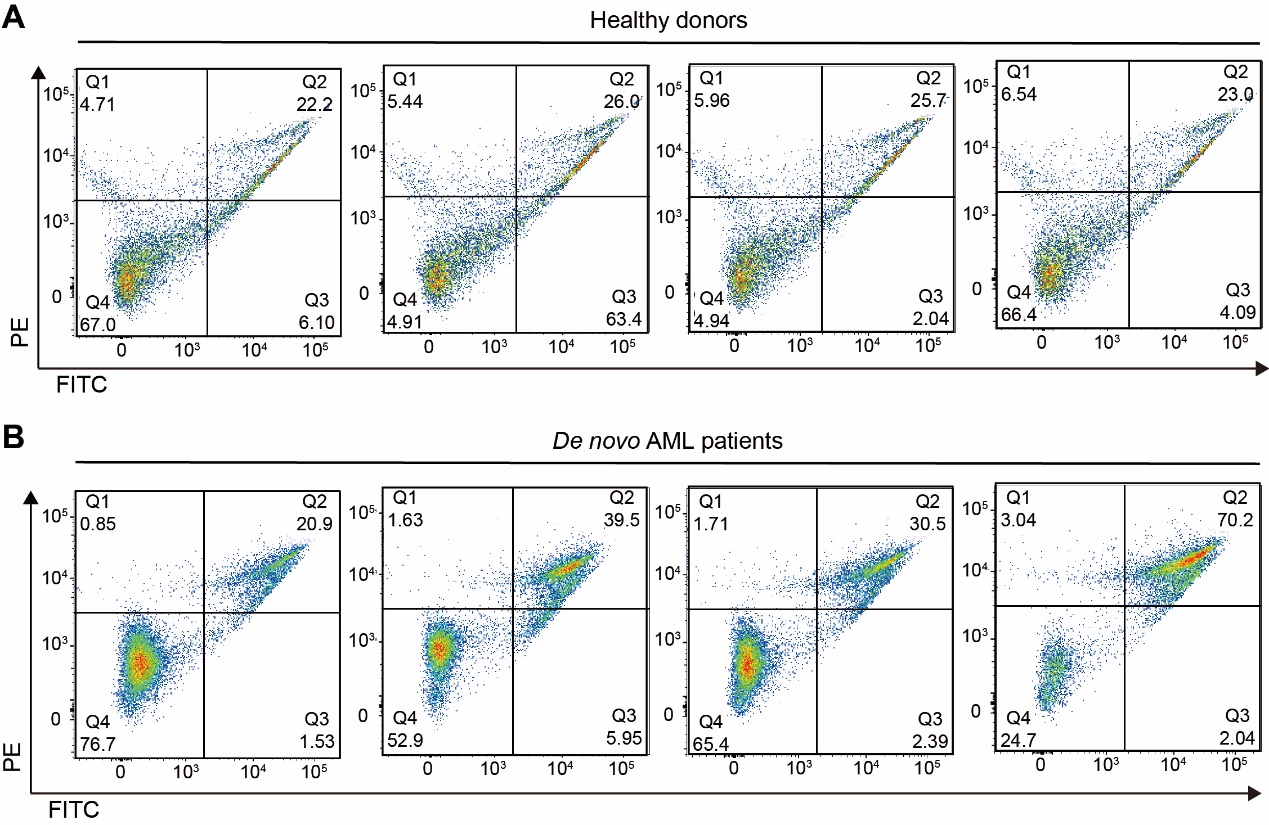

Supplement: Supplementary file 1 [file Table_1.docx]
